# Cytology interpretation after a change to HPV testing in primary cervical screening: Observational study from the English pilot

**DOI:** 10.1002/cncy.22572

**Published:** 2022-04-04

**Authors:** Matejka Rebolj, Christopher S. Mathews, Karin Denton, for the HPV Pilot Steering Group, Tracey‐Louise Appleyard, Margaret Cruickshank, Kate Cuschieri, Kay Ellis, Chris Evans, Viki Frew, Thomas Giles, Alastair Gray, Miles Holbrook, Katherine Hunt, Henry Kitchener, Tanya Levine, Emily McBride, David Mesher, Timothy Palmer, Janet Parker, Elizabeth Rimmer, Hazel Rudge Pickard, Alexandra Sargent, David Smith, John Smith, Kate Soldan, Ruth Stubbs, John Tidy, Xenia Tyler, Jo Waller

**Affiliations:** ^1^ Cancer Prevention Group School of Cancer and Pharmaceutical Sciences Faculty of Life Sciences and Medicine King's College London London United Kingdom; ^2^ Severn Pathology Southmead Hospital North Bristol NHS Trust Bristol United Kingdom

**Keywords:** cytology, uterine cervical neoplasms, mass screening, papillomavirus infections

## Abstract

**BACKGROUND:**

Overcalling of abnormalities has been a concern for using cytology triage after positive high‐risk human papillomavirus (HPV) tests in cervical screening.

**METHODS:**

The authors studied the detection of cytological and histological abnormalities at age 24 to 64 years, using data from the English HPV pilot. The pilot compared routine implementation of primary cervical screening based on cytology (N = 931,539), where HPV test results were not available before cytology reporting, with that based on HPV testing (N = 403,269), where cytology was only required after positive HPV tests.

**RESULTS:**

Revealed HPV positivity was associated with a higher direct referral to colposcopy after any abnormality (adjusted odds ratio [OR_adj_], 1.16; 95% confidence interval [CI], 1.14‐1.18). Laboratories with higher direct referral referred fewer persistently HPV‐positive women after early recall. The detection of high‐grade cervical intraepithelial neoplasia (CIN2+) after direct referral increased with an OR_adj_ of 1.17 (95% CI, 1.13‐1.20) for informed versus uninformed cytology. Generally, the positive predictive value (PPV) of colposcopy for CIN2+ remained comparable under both conditions of interpreting cytology. In women 50 to 64 years old with high‐grade dyskaryosis, however, the PPV increased from 71% to 83% after revealing HPV positivity (OR_adj_, 2.05; 95% CI, 1.43‐2.93).

**CONCLUSIONS:**

Quality‐controlled cervical screening programs can avoid inappropriate overgrading of HPV‐positive cytology.;

## Abstract 2

These population‐based data show that the interpretation of triage cytology informed by a positive human papillomavirus (HPV) test can contribute to a higher overall detection of high‐grade cervical intraepithelial neoplasia without a concomitant increase in overreferral of low‐risk women. The practices used in England, where the national cervical screening program is quality‐assured using rigorous national standards, may serve as a model for screening settings where revealing HPV positivity has led to overgrading of cytology.

## Introduction

Women who are high‐risk human papillomavirus (HPV)‐positive in a cervical screening context are generally triaged given the transience of most HPV infections. One of the most common triage tests internationally is cytology. In randomized trials, where cytology slides were prepared for all women and interpreted without knowledge of the HPV test result, cytology triage showed both safety and efficiency in preventing unnecessary colposcopy referral and biopsies.^1^, ^2^ However, for routine screening outside a trial setting, cytology slides are prepared only after a positive HPV test. Because cytology relies on clinical interpretation of cellular changes, information on HPV positivity may make the screener more attentive to true abnormalities that would have otherwise been missed or dismissed. It could, however, also lead to overcalling of clinically insignificant cellular changes.

In a number of studies, cytological abnormalities were reported more frequently after HPV positivity had been revealed,^3^, ^4^, ^5^, ^6^, ^7^, ^8^, ^9^, ^10^, ^11^ demonstrating the potential for an increased colposcopy referral. A few studies also showed that some of the extra cytological abnormalities detected with “informed” cytology were in women who had high‐grade cervical intraepithelial neoplasia (CIN2+) detected at colposcopy. However, these studies could not provide definitive data on either the CIN2+ detection or the positive predictive value (PPV) of a colposcopy because they tended to be undertaken in experimental settings (eg, as reviews of archived cytology slides). Thus, elucidating the effect of informed cytology on the overall performance of HPV‐based cervical screening requires further investigation.

**Figure 1:**
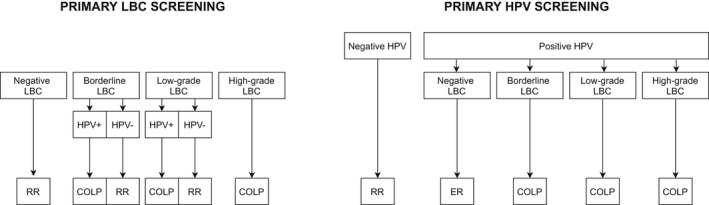
Clinical management of women screened in the English cervical screening program and the pilot study. COLP indicates direct referral to colposcopy; ER, early recall at 12 months, which includes a colposcopy for women with persistently positive HPV tests and incident LBC abnormalities, and a 24‐month early recall for other women with persistently positive HPV tests; HPV, human papillomavirus tests; LBC, liquid‐based cytology; RR, routine recall (ie, a new invitation in 3 or 5 years depending on the woman's age).

The English HPV screening pilot was undertaken within the quality‐controlled cervical screening program (CSP).^12^ Six laboratories implemented HPV‐based screening in parallel with liquid‐based cytology (LBC) screening. With LBC screening, samples were sent for HPV triage only after cytology had been reported. With HPV screening, all triage cytology was from women with positive HPV tests. Thereafter, the women's clinical management depended on the grade of their abnormalities (Fig. 1).

Using these data, we studied how cytology reporting and the downstream consequences thereof were affected according to whether a woman's positive HPV test was unknown (LBC screening) or revealed (HPV screening).

## Materials and Methods

### The Study Setting

The CSP routinely recalls women 25 to 49 years old every 3 years and those 50 to 64 years old every 5 years. The first invitation is sent at 24.5 years. Abnormal cytology in squamous or endocervical cells is defined as borderline changes or worse (Supporting Table 1). The program follows published quality assurance guidelines.^13^

For the pilot, 6 CSP laboratories partially converted to HPV‐based screening and continued to provide LBC screening to the remainder of their catchment areas.^12^, ^14^, ^15^ In all laboratories, the same staff handled cytology slides from both HPV‐based and LBC screening. The baseline testing in the first screening round started between May and August 2013, depending on the laboratory, and was completed by the end of December 2016. Data continued to be collected for any follow‐up tests. Most women involved had been invited by the program previously and thus were likely to have undergone LBC screening. It is unlikely that they had been previously screened with HPV testing because this was not offered routinely in England. Because HPV vaccine eligible cohorts in the United Kingdom represent those born in or after 1990, by far the majority of women included in this analysis would not have been eligible for HPV vaccination routinely. We estimated, using national vaccination coverage data,^16^ that 94% of women screened in the pilot at younger than 30 years old had not been vaccinated. The remaining 6% were vaccinated while they were eligible to receive the vaccine through the national catch‐up campaign at 16 to 17 years old. Older women undergoing screening in 2013 to 2016 were unlikely to have been vaccinated.^17^, ^18^

The laboratories used SurePath (BD, Sparks, MD) and ThinPrep (Hologic, Marlborough, MA) LBC systems (Supporting Table 2). HPV testing was undertaken using cobas 4800 (Roche, Rotkreuz, Switzerland), RealTime (Abbott, Wiesbaden, Germany), APTIMA (Hologic), and, for a smaller component of tests, Hybrid Capture 2 (Qiagen, Gaithersburg, MD) tests.

In LBC screening, women with high‐grade dyskaryosis and those with borderline changes or low‐grade dyskaryosis combined with a positive HPV test were directly referred to colposcopy; other women were returned to age‐appropriate routine recall (Fig. 1). In HPV‐based screening, women with a positive HPV test showing borderline cytological changes or worse were directly referred to colposcopy. Women with a positive HPV test and negative cytology were retested in an early recall at 12 months and were referred to colposcopy if they remained HPV‐positive and had an incident cytological abnormality, or, in 3 laboratories, if they showed a persistent HPV16/18 infection combined with negative cytology.^15^ Women with persistently positive HPV tests who were not referred at the 12‐month early recall were retested again at 24 months from baseline and referred to colposcopy if they remained HPV‐positive. Women with negative HPV tests at baseline or any of the 2 early recalls were returned to routine recall.

Screening and colposcopy data until the end of 2019 with dates, types of tests, and diagnoses were retrieved from the laboratory information systems. Information on cervical cancer diagnoses until the end of 2018 was retrieved from the English National Cancer Register.^19^ The unique English National Health Service (NHS) numbers were used for linkage.

### Statistical Analysis

We excluded women who had recent cervical abnormalities or any cervical cancer diagnosis before their first pilot test in 2013 to 2016, because those tests were likely made in response to earlier abnormalities. The remaining tests were considered to be routine primary screening tests. We also excluded women without a definitive diagnosis on screening and/or triage tests (0.7% for LBC and 0.2% for HPV testing).

We first studied whether revealing the positive HPV test result affected cytology interpretation. For both HPV‐based and LBC screening, we calculated age‐specific proportions of women with abnormal cytology requiring direct colposcopy referral after the baseline sample (Fig. 1). Because the English CSP does not automatically cotest all samples for HPV and cytology, HPV screening algorithm no longer identifies HPV‐negative women with high‐grade dyskaryosis. Thus, under the assumption that revealed HPV positivity does not affect cytology interpretation, HPV‐based screening should result in a slightly lower proportion of directly referred women than LBC screening. Abnormal cytology was stratified into borderline changes, low‐grade dyskaryosis, and high‐grade dyskaryosis. Age was categorized as 24 to 29, 30 to 49, and 50 to 64 years. Logistic regression odds ratios (OR) for informed cytology (in triage after primary HPV‐based screening) versus uninformed cytology (as the primary screening test) were adjusted for women's age, laboratory as a proxy for unmeasured local characteristics, and decile of index of multiple deprivation (IMD), which is a standard English area‐based measure of deprivation.^20^ Higher IMD deciles are associated with lower deprivation.

Thereafter, the data were stratified by laboratory, and, within each laboratory, into consecutive 3‐month periods (ie, calendar quarters). For each laboratory and calendar quarter, we calculated the ratio of the proportions with a direct colposcopy referral after informed versus uninformed cytology. These ratios were standardized for age (<30 and ≥30 years) and IMD decile (deciles 1‐5 vs deciles 6‐10) to represent the female population of England in 2013.^21^ If a laboratory was initially reporting more abnormalities with informed than with uninformed cytology (ie, exhibited a ratio higher than 1), we assumed that a downward trend toward the value of 1 would be indicative of a reduction in overgrading of cytology in women with positive HPV tests (learning curve).

We then investigated the downstream consequences of revealing the HPV infection. First, we studied the detection of CIN2+ after direct colposcopy referral for informed versus uninformed cytology and the associated PPV for CIN2+ of those colposcopies. ORs were calculated and adjusted as described above. For women with positive primary HPV tests, we also stratified these data by LBC system (ThinPrep and SurePath). Here, we estimated the sensitivity, specificity, PPV, and the negative predictive value (NPV) for CIN2+ of baseline triage cytology with exact binomial 95% confidence intervals (CI). CIN2+ were counted until the end of the episode, including both early recalls for women with initially negative triage cytology; cases diagnosed at early recall were considered missed by baseline triage cytology. ORs for the observed accuracy measures comparing ThinPrep with SurePath were adjusted for age, IMD decile, and HPV test type used in the laboratory (ie, detecting viral DNA vs mRNA). Second, we studied the relationship between direct referral and early recall referral. For each laboratory and calendar quarter, the proportions of women with direct referral were calculated as described above, using the numbers with a positive HPV test as the denominator. The proportions of women with a positive HPV test, screened in the same laboratory and calendar quarter, who satisfied the criteria for colposcopy referral at early recall were calculated separately. Total referral was defined as the sum of direct and early recall referral out of all women with a positive HPV test in that laboratory and calendar quarter. All proportions were standardized to the English population as described above. R version 3.6.1 was used for analyses.

## Results

### Changes in Cytological Interpretation

The study included 931,539 women screened with LBC and 403,269 women screened with HPV testing (Table 1). Among these, 3.6% and 4.0%, respectively, were directly referred to colposcopy after abnormal cytology (OR_adj_ for informed vs uninformed cytology, 1.16; 95% CI, 1.14‐1.18). Across all age groups, the increase in referrals associated with informed reading was highest for borderline changes (OR_adj_, 1.35; 95% CI, 1.30‐1.40). The increase in low‐grade dyskaryosis was smaller and limited to women younger than 50 (with OR_adj_ consistently approximately 1.15). Although the reporting of high‐grade dyskaryosis with informed reading only showed a very small increase in women younger than 30, it was significantly less frequent among women older than 50 (OR_adj_, 0.81; 95% CI, 0.71‐0.93).

**TABLE 1:** Detection of Cytological Abnormalities and CIN2+ for Informed (Primary HPV‐Based Screening) and Uninformed (Primary LBC Screening) Cytology Interpretation by Grade of Cytological Abnormality and Age Group

Age (y)	Reported Abnormal Cytology in the Screening Sample			Detection of CIN2+ After a Cytologically Abnormal Screening Sample		
	Informed, No. (Per 100 Screened)	Uninformed, No. (Per 100 Screened)	OR_adj_ for Informed vs Uninformed (95% CI)	Informed, No. (Per 1000 Screened)	Uninformed, No. (Per 1000 Screened)	OR_adj_ for Informed vs Uninformed (95% CI)
24‐29						
N screened	76,096	177,502		76,096	177,502	
Borderline	2216 (2.9%)	3907 (2.2%)	1.30 (1.23‐1.37)	538 (7.1‰)	876 (4.9‰)	1.38 (1.24‐1.54)
Low‐grade	2931 (3.9%)	6165 (3.5%)	1.14 (1.09‐1.19)	591 (7.8‰)	1018 (5.7‰)	1.37 (1.23‐1.52)
High‐grade	2755 (3.6%)	6016 (3.4%)	1.07 (1.02‐1.12)	2406 (31.6‰)	5154 (29.0‰)	1.10 (1.04‐1.15)
Total	7902 (10.4%)	16,088 (9.1%)	1.16 (1.13‐1.20)	3535 (46.5‰)	7048 (39.7‰)	1.18 (1.13‐1.23)
30‐49						
N screened	224,037	524,824		224,037	524,824	
Borderline	2122 (0.9%)	3721 (0.7%)	1.37 (1.30‐1.45)	419 (1.9‰)	687 (1.3‰)	1.42 (1.26‐1.61)
Low‐grade	2682 (1.2%)	5767 (1.1%)	1.16 (1.11‐1.21)	430 (1.9‰)	695 (1.3‰)	1.53 (1.36‐1.73)
High‐grade	2248 (1.0%)	5382 (1.0%)	1.00 (0.95‐1.05)	1932 (8.6‰)	4347 (8.3‰)	1.07 (1.01‐1.13)
Total	7052 (3.1%)	14,870 (2.8%)	1.16 (1.13‐1.19)	2781 (12.4‰)	5729 (10.9‰)	1.17 (1.11‐1.22)
50‐64						
N screened	103,136	229,213		103,136	229,213	
Borderline	447 (0.4%)	713 (0.3%)	1.46 (1.30‐1.65)	47 (0.5‰)	92 (0.4‰)	1.20 (0.84‐1.72)
Low‐grade	482 (0.5%)	1028 (0.4%)	1.09 (0.98‐1.22)	37 (0.4‰)	75 (0.3‰)	1.12 (0.75‐1.67)
High‐grade	286 (0.3%)	810 (0.4%)	0.81 (0.71‐0.93)	229 (2.2‰)	522 (2.3‰)	1.00 (0.86‐1.18)
Total	1215 (1.2%)	2551 (1.1%)	1.11 (1.03‐1.19)	313 (3.0‰)	689 (3.0‰)	1.04 (0.91‐1.19)
24‐64						
Total screened	403,269	931,539		403,269	931,539	
Borderline	4785 (1.2%)	8341 (0.9%)	1.35 (1.30‐1.40)	1004 (2.5‰)	1655 (1.8‰)	1.39 (1.28‐1.51)
Low‐grade	6095 (1.5%)	12,960 (1.4%)	1.15 (1.11‐1.18)	1058 (2.6‰)	1788 (1.9‰)	1.42 (1.32‐1.54)
High‐grade	5289 (1.3%)	12,208 (1.3%)	1.02 (0.99‐1.06)	4567 (11.3‰)	10,023 (10.8‰)	1.08 (1.04‐1.12)
Total	16,169 (4.0%)	33,509 (3.6%)	1.16 (1.14‐1.18)	6629 (16.4‰)	13,466 (14.5‰)	1.17 (1.13‐1.20)

Abbreviations: CI, confidence interval; CIN2+, cervical intraepithelial neoplasia grade 2 or higher; HPV, high‐risk human papillomavirus; LBC, liquid‐based cytology; OR_adj_, odds ratio, adjusted for women's age (in years), laboratory, and decile of Index of Multiple Deprivation.

**Figure 2:**
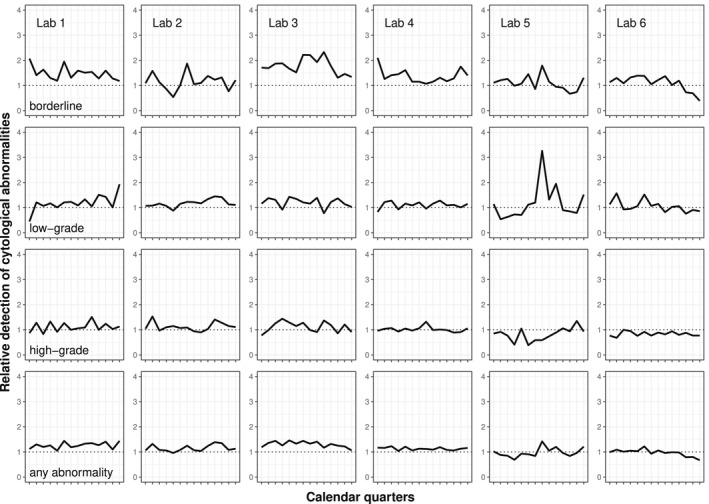
Temporal trends in the ratios for the reporting of cytological abnormalities comparing informed (human papillomavirus [HPV]‐based primary screening) versus uninformed (liquid‐based cytology [LBC] primary screening) cytology interpretation, by cytological grade and laboratory. Note that relative detection was calculated for each laboratory, cytological grade, and calendar quarter including the period between the third quarter of 2013 and end of 2016 as: (the proportion of women with abnormalities after informed cytology in HPV‐based primary screening)/(the proportion of women with abnormalities after uninformed cytology in LBC primary screening). Dashed line represents the point where the ratio equals 1.

Figure 2 reports the ratios of standardized proportions of women directly referred to colposcopy comparing informed with uninformed cytology. As in Table 1, these ratios tended to be higher for borderline than for more severely abnormal cytology. However, they differed between the laboratories. Overall, laboratories 1 through 4 tended to have ratios higher than 1 indicating a higher frequency of reporting abnormalities associated with informed cytology. Overall, the ratios remained above 1 throughout the pilot's first screening round. In laboratories 5 and 6, the ratios tended to be lower for all cytological grades than in laboratories 1 through 4. There were few time‐limited exceptions (eg, the very high ratio for low‐grade dyskaryosis in laboratory 5 in 1 calendar quarter that was due to a drop in the reporting of abnormal uninformed cytology whereas the reporting of abnormal informed cytology remained similar as in the adjacent quarters).

### Effect on CIN2+ Detection

Overall, informed reading was associated with a higher detection of CIN2+ at direct referral for all abnormal cytology grades combined (OR_adj_, 1.17; 95% CI, 1.13‐1.20) (Table 1). This was particularly pronounced for women younger than 50 with borderline or low‐grade abnormalities. In women older than 50, CIN2+ detection was similar for informed and uninformed cytology, even among those with high‐grade dyskaryosis (OR_adj_, 1.00; 95% CI, 0.86‐1.18).

Informed cytology was associated with a slightly higher PPV of baseline colposcopies for CIN2+, but this varied by age group (Table 2). Among women older than 50 with high‐grade dyskaryosis, the PPV increased from 71% with uninformed to 83% with informed cytology (OR_adj_, 2.05; 95% CI, 1.43‐2.93). For all ages combined, 4.5 women with informed borderline cytology needed to be referred to detect one CIN2+. This varied between 4.0 among women younger than 30 to 9.1 among women older than 50. Among women with low‐grade abnormalities detected through informed cytology, 5.5 needed to be referred overall to detect 1 CIN2+, varying between 4.8 and 12.5 depending on the women's age. Among those with high‐grade abnormalities, 1.1 women needed to be referred to detect 1 CIN2+, varying between 1.1 and 1.2 depending on the women's age.

**TABLE 2:** PPV for CIN2+ After Direct Colposcopy Referral for Informed (Primary HPV‐Based Screening) and Uninformed (Primary LBC Screening) Cytology Interpretation by Grade of Cytological Abnormality Reported for the Screening Sample and Age Group

Age (y)	Cytology Interpretation		
	Colposcopies (PPV %)		OR_adj_ for Informed vs Uninformed (95% CI)
	Informed	Uninformed	
24‐29			
Borderline	2110 (25)	3699 (24)	1.11 (0.98‐1.26)
Low‐grade	2781 (21)	5775 (18)	1.26 (1.12‐1.41)
High‐grade	2678 (90)	5701 (90)	0.97 (0.83‐1.13)
30‐49			
Borderline	2041 (21)	3499 (20)	1.05 (0.91‐1.20)
Low‐grade	2555 (17)	5344 (13)	1.36 (1.19‐1.56)
High‐grade	2188 (88)	5045 (86)	1.20 (1.03‐1.40)
50‐64			
Borderline	423 (11)	666 (14)	0.83 (0.56‐1.22)
Low‐grade	450 (8)	967 (8)	1.08 (0.71‐1.64)
High‐grade	277 (83)	734 (71)	2.05 (1.43‐2.93)
24‐64			
Borderline	4574 (22)	7864 (21)	1.07 (0.97‐1.17)
Low‐grade	5786 (18)	12,086 (15)	1.29 (1.18‐1.40)
High‐grade	5143 (89)	11,480 (87)	1.14 (1.03‐1.27)

Abbreviations: CI, confidence interval; CIN2+, cervical intraepithelial neoplasia grade 2 or higher; HPV, high‐risk human papillomavirus; LBC, liquid‐based cytology; OR_adj_, odds ratio, adjusted for women's age (in years), laboratory, and decile of Index of Multiple Deprivation; PPV, positive predictive value.

Numbers of CIN2+ lesions are reported in Table 1.

Although ThinPrep LBC sites had slightly lower HPV positivity in primary screening than SurePath sites (11.7% vs 12.2%; OR_adj_, 0.89; 95% CI, 0.87‐0.91), they had similar proportions of positive HPV tests followed by abnormal cytology triage (34.5% vs 32.9%; OR_adj_, 1.04; 95% CI, 1.00‐1.09; *P* = .06; not tabulated). In ThinPrep sites, baseline triage cytology after a positive HPV test detected 69% (1842/2686) of all CIN2+, whereas in SurePath sites this was 73% (4787/6556) (OR_adj_, 0.83; 95% CI, 0.75‐0.93) (Table 3). The specificity and the PPV were also somewhat lower in ThinPrep than in SurePath sites.

**TABLE 3:** Detection of CIN2+ Among Women With Positive HPV Tests With Informed Triage Cytology by Brand of LBC and the Outcome of Cytology Triage at Baseline

Baseline Cytology Outcome	ThinPrep		SurePath		ThinPrep vs SurePath
	CIN2+	<CIN2	CIN2+	<CIN2	OR_adj_ (95% CI)^a^
Test+	1842	3364	4787	6176	
Test−	844	9046	1769	20,552	
Sensitivity^b^	69% (67‐70)		73% (72‐74)		0.83 (0.75‐0.93)
Specificity^c^	73% (72‐74)		77% (76‐77)		0.83 (0.79‐0.88)
NPV^d^	91% (91‐92)		92% (92‐92)		1.00 (0.91‐1.10)
PPV^e^	35% (34‐37)		44% (43‐46)		0.70 (0.65‐0.76)

Abbreviations: A, test+/CIN2+; B, test−/CIN2+; C, test+/<CIN2; CI, confidence interval; CIN, cervical intraepithelial neoplasia; D, test−/<CIN2; HPV, high‐risk human papillomavirus; LBC, liquid‐based cytology; NPV, negative predictive value; OR_adj_, adjusted odds ratio.

In total, 129,404 women were screened in ThinPrep sites, and 273,865 were screened in SurePath sites. For proportions, numbers in parentheses are exact binomial 95% CIs.

^a^ Adjusted for age in years, decile of the Index of Multiple Deprivation, and the type of HPV test used in the laboratory.

^b^ Sensitivity = A/(A + B).

^c^ Specificity = D/(C + D).

^d^ NPV = D/(B + D).

^e^ PPV = A/(A + C).

### Direct, Early Recall, and Total Colposcopy Referral

**Figure 3:**
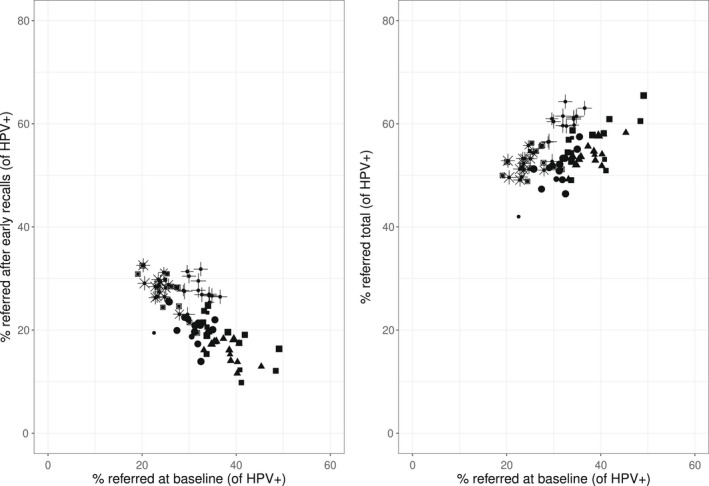
The relationship between the proportions of women with a positive human papillomavirus (HPV) test who were referred to colposcopy directly after the baseline test and those who were referred after early recall and between the proportions of women with a positive HPV test who were referred directly and those who were referred either directly or after early recall (total referral), by laboratory and calendar quarter. Note that each unit represents a specific laboratory in a specific calendar quarter. The 6 different shapes represent the 6 pilot laboratories. The size of the unit represents the number of HPV‐positive samples that the laboratory handled in a specific calendar quarter.

Figure 3 shows that the laboratories with higher direct colposcopy referral tended to refer fewer women after early recall. The relationship was also observed within laboratories. In each laboratory, calendar quarters with higher direct referral tended to show lower early recall referral, compared to quarters with lower direct referral. Overall, this means that the increase in total referral was mitigated by a lower early recall referral.

## Discussion

### Main Findings

These population‐based data from routine implementation of cervical screening in more than 1.3 million women showed that cytology informed by HPV positivity was associated with a 35% increase in the reporting of borderline changes and a 15% increase in the reporting of low‐grade dyskaryosis. Although this resulted in an increased colposcopy referral, the PPV for CIN2+ did not decline.

### Strengths and Weaknesses

Apart from its size, the strength of our study is that the same laboratory personnel reported both informed and uninformed cytology in a national quality‐assured screening program with exacting training requirements. Women were managed according to predefined protocols, with high levels of adherence.^14^ These data remain representative after the national roll‐out of HPV‐based screening, because the CSP continues to use the same quality assurance and clinical management protocols and the same testing platforms. A relative weakness of this study is that screening tests were not allocated in a random process. However, all comparisons between the 2 screening methods were adjusted for age and deprivation, the 2 principal factors associated with screening attendance and detection of abnormalities.^22^, ^23^

### Clinical Implications and Comparison With the Literature

Because colposcopy referral induces anxiety^24^ and increases health care resource use, referrals need to be targeted to include women with the highest risk of an underlying CIN2+ lesion. With cytology informed by HPV positivity, the main concern was the potential for an increased referral of low‐risk women. Our data are reassuring in this regard. Although more women younger than 50 were directly referred to colposcopy following informed abnormal cytology, their colposcopies were just as likely to result in a diagnosis of CIN2+ as colposcopies following uninformed cytology. In women older than 50, the most interesting finding was a reduction in referral following high‐grade dyskaryosis resulting in an increased PPV for CIN2+ and the same detection of CIN2+ as with uninformed cytology. It appears that when aware of a positive HPV test, cytoscreeners are better able to differentiate between abnormalities associated with the development of cervical cancer and abnormalities associated with hormone‐related aging artifacts such as atrophy. Consistent with an Italian study,^25^ our data suggest that some of the additional direct colposcopy referrals after informed cytology may reflect referrals that would have been indicated at a later time point. An earlier referral, as compared with a delayed referral, has indirect clinical benefits for women with CIN2+ such as a reduced risk of nonadherence. Among women in the pilot for whom a referral decision was delayed to early recall, and who were followed up for 3 to 5.5 years, ~15% did not attend testing at 1 or both recalls and a further 5% to 10% of those with persistently positive tests did not undergo a colposcopy.^14^ With direct referral, only ~3% of all referred women did not undergo a colposcopy.

The context of reading cytology informed by HPV positivity is similar to that of an unblinded slide review for women who developed cervical cancer. In the English CSP, false‐negative cytology appears to explain fewer than 3% of all cervical cancer cases.^26^ The upgrading of negative to abnormal cytology after unblinded review is most frequently made for recent slides^27^ and those often contain the same HPV genotypes as the cancer tissue.^28^ These findings further reinforce the impression from our study that paying attention to the existing HPV infection while interpreting cytology may enhance prevention of cervical cancer through an earlier detection of progressive CIN2+.

Although this did not appear to negatively affect screening outcomes at the aggregate level, we nevertheless observed some local variation in the patterns of reporting informed cytology. Similar variations between screening units have been reported previously. In 10 Italian centers, the proportions of women with positive HPV tests whose triage cytology was reported as abnormal varied between 20% and 57%.^25^ In a US study, informed cytology increased the detection of CIN2+ in 2 out of 4 laboratories.^5^ We could not find a satisfactory explanation for why we saw a variation in the English pilot study. There was no consistent pattern that would separate the laboratories with excess reported abnormalities from those without such an excess. The 6 laboratories used different screening technologies and had different working practices (eg, whether they employed checkers). Previous studies comparing cytology interpretation in all CSP laboratories found some differences but few true outliers with respect to the correlation between cytology interpretation and biopsy results.^29^ It is thought that the underlying differences in cytology reporting could be partially explained by population characteristics in the respective catchment areas,^30^ although local variation in colposcopy provision might also play a role. To standardize the practice across the program, all staff undertake mandatory external quality assurance, and the whole CSP is carefully quality assured with reference to standards required for both process and outcome. All laboratories are staffed broadly in line with the British Association of Cytopathology code of practice,^31^ which requires a minimum number of cytology slides to be read per laboratory each year (35,000), defines staffing roles and responsibilities, limits the number of hours performing screening tasks (to 5 per day), defines recommended breaks during the working day, etc. The laboratories, furthermore, need to achieve a 14‐day turnaround time for the reporting of the results. This allows them approximately 10 days for all laboratory processes including HPV testing and cytology. Although there is daily variation in the received workload, this can be smoothed over several days. All cytoscreeners undergo a 2‐year mandatory training program in addition to their other qualifications, whereas pathologists must complete a specialized training in cervical cytology. All staff undergo mandatory update training every 3 years provided by approved training providers, ensuring awareness of new developments and of any areas which have been found to cause problems anywhere in the CSP. By submitting all samples to a second, rapid, review, cytoscreeners are monitored for the sensitivity of abnormality detection, whereas pathologists are monitored for specificity. All outcome measures are published to allow comparison between laboratories^32^ and any outliers are fully investigated. Within the pilot, the largest differences between informed and uninformed cytology were found for borderline changes; according to the British cytology morphology criteria, these changes are a positive finding and not an expression of uncertainty.^33^ The staff providing cytology interpretation in the pilot study were instructed to follow these morphological criteria whether or not the slides were from women with positive HPV tests, and this continues to be the case at present.^34^

A large English randomized trial (Manual Assessment Versus Automated Reading In Cytology) reported no differences in the detection of CIN2+ between the SurePath and ThinPrep systems in primary LBC screening.^35^ Danish and Dutch studies using routinely collected data, however, showed more pronounced differences including a lower incidence of cervical cancer 6 years after a negative SurePath test compared with a negative ThinPrep test (adjusted hazard radio, 0.71; 95% CI, 0.58‐0.87).^36^, ^37^ When used for triage of women with positive HPV tests in the English CSP, the consequences of false‐negative cytology are less profound because negative cytology is no longer a condition for a definitive return to routine recall. In our study, the differences between the 2 LBC systems used in triage were relatively small. Both ThinPrep and SurePath delayed the diagnosis of CIN2+ to early recall after negative baseline triage cytology in approximately 30% of the cases.

In conclusion, implementation of HPV‐based primary screening with cytology triage in a controlled manner, supported by rigorous cytology training and performance monitoring, does not lead to inappropriate overgrading of cytology. Rather, an earlier recognition of cytological abnormalities appears to partly explain why the detection of CIN2+ is increased in HPV‐based screening compared with LBC screening.

## Funding Support

Public Health England supported the epidemiological evaluation of the HPV pilot (ODR1718_428). Matejka Rebolj and Christopher Mathews were supported by Cancer Research UK (C8162/A27047). Public Health England had a role in designing the pilot and in the collection of the data and commented on the manuscript. Cancer Research UK had no role in designing the study, in the collection of the data, or in the writing of the manuscript.

## Conflict of Interest Disclosures

Christopher S. Mathews held an honorary appointment at Public Health England to process the data for the pilot. Karin Denton reports personal fees from Public Health England during the conduct of the study and travel support from Hologic outside the submitted work; chairs the Public Health England Laboratory Clinical Professional Group, the HPV Development Group, and several groups related to the evaluation of self‐sampling; was a consultant to the Scally Review of cervical screening in Ireland and the Royal College of Obstetricians and Gynaecologists review of cervical cancer audit in Ireland (both completed in 2019); and has prepared expert medicolegal reports for claimants and defendants, including cases of cervical cancer. Matejka Rebolj reports grants from Public Health England during the conduct of the study and lecture fees from Hologic outside the submitted work, is a member of the Public Health England Laboratory Technology Group and HPV Self‐Sampling Operational Steering Group and Project Board, and has attended meetings with various human papillomavirus assay manufacturers.

## Author Contributions

**Matejka Rebolj:** Conceptualization, methodology, formal analysis, writing–original draft, and writing–review and editing. **Christopher S. Mathews:** Data management and writing–review and editing. **Karin Denton:** Conceptualization, methodology, and writing–review and editing. The Pilot Steering Committee was responsible for the study design of the pilot.

## Supporting information

Supplementary Material

## Data Availability

The data in this article belongs to the former Public Health England and the authors cannot provide access to the relevant data sets to third parties. Requests for data and pre‐application advice should instead be made to Office for Data Release (ODR@phe.gov.uk).
