# Supplementary material for: Cytology interpretation after a change to HPV testing in primary cervical screening: Observational study from the English pilot
Source: Cancer Cytopathol. 2022 Apr 4;130(7):531–41. doi: 10.1002/cncy.22572 (PMC9542289; doi:10.1002/cncy.22572)
Supplement: Supplementary file 1 — Supplementary Material [file CNCY-130-531-s001.docx]

Cancer Prevention Group

School of Cancer & Pharmaceutical Sciences

Faculty of Life Sciences & Medicine

King’s College London

London, 24 January 2022

**CYTOLOGY INTEPRETATION AFTER A CHANGE TO HUMAN PAPILLOMAVIRUS TESTING IN PRIMARY CERVICAL SCREENING:**

**OBSERVATIONAL STUDY FROM THE ENGLISH PILOT**

**SUPPLEMENTARY INFORMATION**

Matejka Rebolj,^a§^ Christopher S Mathews,^a^  Karin Denton,^b^

on behalf of the HPV pilot steering group

^a^ Cancer Prevention Group, School of Cancer & Pharmaceutical Sciences, Faculty of Life Sciences & Medicine, King’s College London, London, UK

^b^ Severn Pathology, Southmead Hospital, North Bristol NHS Trust, Bristol, UK

^§^ Corresponding author, matejka.rebolj@kcl.ac.uk

Summary. This file contains a comparison between the UK and the Bethesda 2014 cytology classifications, and a table with the numbers of women participating in the pilot study by laboratory site and primary screening test.

Table S1. Comparison between the cytological terminology recommended by the British Society for Clinical Cytology (revised in 2008) and the Bethesda 2014 classification. Adapted from Denton et al. (Cytopathology 2008;19:137-157)

| **Bethesda 2014** | **BSCC classification** |
| --- | --- |
| Unsatisfactory for Evaluation | Inadequate |
| Negative for Intraepithelial lesion or Malignancy | Negative |
| Abnormal Cells of Undetermined Significance (ASCUS) |  |
|  | Borderline change in squamous cells |
| Abnormal Cells of Undetermined Significance, Cannot Exclude HSIL (ASC-H) |  |
| Low Grade Squamous Intraepithelial Lesion (LSIL) | Low Grade Dyskaryosis |
| High Grade Squamous intraepithelial lesion (HSIL) | High Grade Dyskaryosis (Moderate) |
|  | High Grade Dyskaryosis (Severe) |
| Squamous Cell Carcinoma | High Grade Dyskaryosis  ?Invasive Squamous carcinoma |
| Atypical Glandular cells: |  |
| Endocervical NOS | Borderline change in endocervical cells^a^ |
| Endometrial NOS | No equivalent |
| Glandular NOS | Borderline change in endocervical cells^a^ |
| Endocervical favour neoplastic | Borderline change in endocervical cells |
| Glandular cells favour neoplastic | Borderline change in endocervical cells |
| Endocervical Adenocarcinoma in situ | ?Glandular neoplasia of endocervical type |
| Adenocarcinoma: |  |
| Endocervical | ?Glandular neoplasia of endocervical type |
| Endometrial | ? Glandular Neoplasia (non cervical) |
| Extrauterine | ? Glandular Neoplasia (non cervical) |
| NOS | No equivalent |

^a^ Thresholds for abnormality are different and these cases may well be classified as negative in the BSCC system.

Table S2. Liquid-based cytology and human papillomavirus test assays used in pilot laboratories.

| **Laboratory** | **LBC system** | **N screened with LBC** | **HR-HPV test** | **N screened with HR-HPV testing** |
| --- | --- | --- | --- | --- |
| 1 | ThinPrep | 117,072 | APTIMA, HC2^a^ | 50,568 |
| 2 | SurePath | 85,141 | APTIMA | 41,007 |
| 3 | ThinPrep | 153,662 | RealTime | 46,738 |
| 4 | SurePath | 291,252 | RealTime, cobas | 143,172 |
| 5 | ThinPrep | 128,366 | cobas | 32,098 |
| 6 | SurePath | 156,046 | cobas | 89,686 |

Note. The numbers of women screened in each laboratory differ slightly from those reported in other publications, owing to variation in exclusion criteria. Here, women were excluded if they did not have a definitive diagnosis on the primary screening and triage tests e.g., following re-testing due to inadequate tests.

^a^ The laboratory used HC2 for a proportion of primary screening samples in the beginning phase, alternating with APTIMA which became the main HPV test assay for this laboratory for the duration of the pilot.
